# Network meta-analysis, a new statistical technique at urologists' disposal to improve decision making

**DOI:** 10.1590/S1677-5538.IBJU.2018.03.02

**Published:** 2018

**Affiliations:** 1 Universidad del Valle, Cali, Colombia, CO; 2 Urological Research Group, UROGIV, Cali, Colombia, CO

## INTRODUCTION

Systematic reviews have been determined to be fundamental tools for establishing the magnitude of an effect, with adequate rigor, methodology and scientific quality (1-4). A meta-analysis is a statistical analysis used in medical investigation, to synthesize information, and compare at least two interventions at a time, regarding an appropriate investigative question (4). Additionally, the available comparisons have to be made, in at least two studies, between intervention A and B otherwise, it is not possible to make it; nonetheless we lack of studies which make all the possible comparisons feasible nowadays (5).

Due to the lack of direct evidence, tools as network meta-analysis and indirect comparisons have been developed, considering all the available studies, and allowing comparisons regarding a common element, to estimate the effect of an intervention in an indirect way (6, 7). Network meta-analysis has also been called multiple-treatment comparison or mixed-treatment comparison meta-analysis (8).

The aim of this review is to expose the introductory concepts of network meta-analyses, and indirect comparisons.

## WHAT IS A NETWORK META-ANALYSIS ABOUT?

Meta-analysis allows to statistically synthesizing the available evidence of studies about a clear clinical research question from an adequate systematic review (3, 9). Additionally, network meta-analysis is a tool designed to evaluate the effectiveness when comparing different treatments with similar characteristics, which have not been directly compared in a study. This is a very frequent case, given that there are no enough studies making comparisons for every intervention, because of the cost, complexity and ethical components. Unlike the traditional meta-analysis, which summarizes the evidence from experiments that have evaluated the same comparison (Intervention A vs. B), this new tool compares the results of different studies that have a point or a common intervention (8, 10).

Network meta-analyses, as we have previously said, are also known as multiple-treatment comparison meta-analysis or mixed-treatment comparison meta-analysis; they allow elucidating indirect estimates when comparing different treatments. The statistical methods used by the network meta-analysis, such as the Bayesian and the frequentist methods, have been described in detail in other publications (11, 12).

At this time, let me suppose a systematic review that evidences experiments comparing treatment A vs. B and others that compare treatment A vs. C. With a conventional meta-analysis, only these kind of interventions could be compared but it would not be possible to make a comparison of A vs. C, which could be clinically important. In these occasions, the meta-analysis of indirect or network comparisons, would be useful.

Whenever it is possible to find estimates of the effect of both direct comparisons and indirect comparisons, the information gathered could increase the precision and power of the effect estimate (8, 13). Indirect comparisons require to establish concepts such as: *transitivity and consistency* that I will explain later (6).

### The geometry of the network

The graphic representation of the network will play a fundamental role in the transparency of the results and in the critical reading of it. This allows us to understand in one way or another, the strength of the evidence, the number of articles from which the information presented comes from (treatment nodes), the comparisons that have direct comparisons and those that present indirect or mixed comparisons and the number of patients with different comparisons, in such a way that confidence in the results is increased (8, 13, 14).

**Figure 1:**
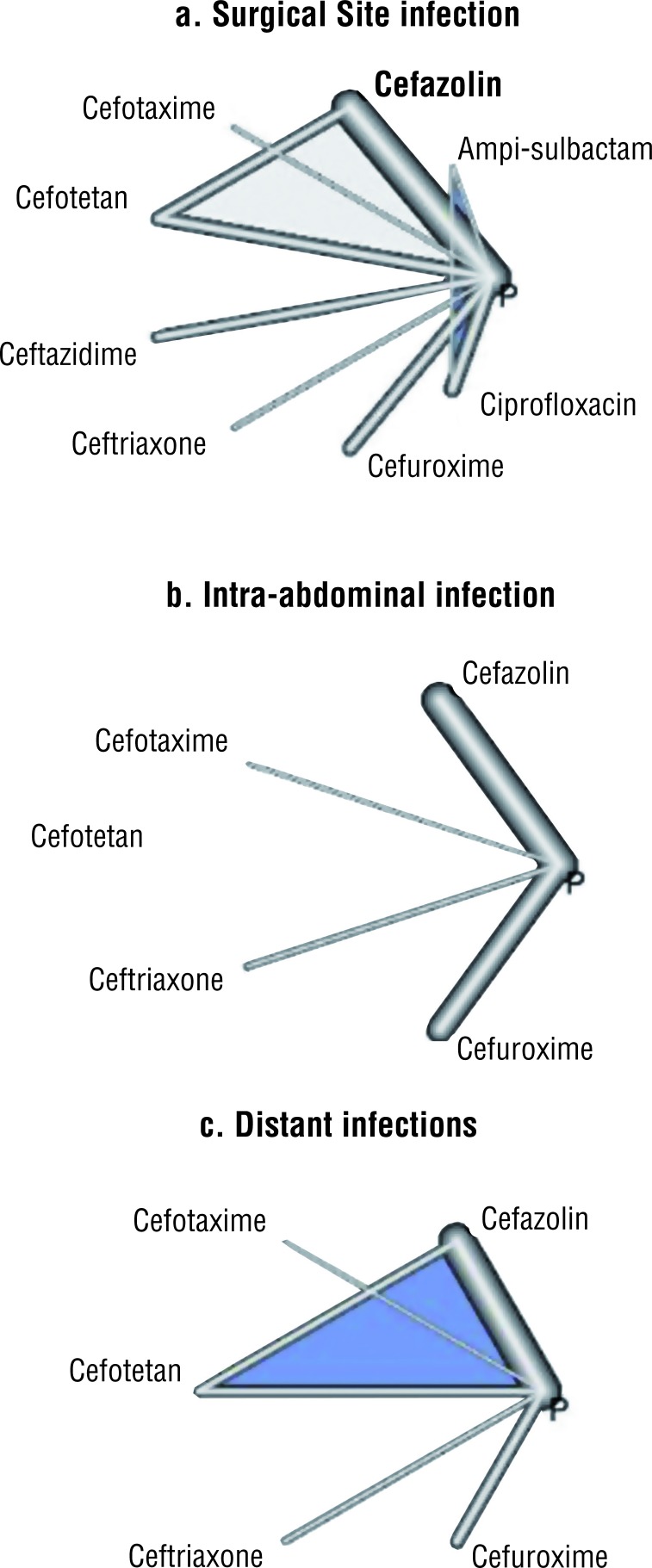
Examples of the geometry of the network.

When there is no evidence of union between two pairs of nodes, it means that no clinical experi-ments were identified for those specific treatments. There are different geometric shapes, depending on the clinical conditions that were studied. For example, if all the experiments are compared with placebo, the geometry will be of a star, on the other hand, if all the interventions are compared with the others, then it will be in the form of a polygon (14) (Figure-1).

### Concepts of validity of network meta-analysis: transitivity and consistency

For indirect or mixed comparisons, the studies must be comparable in terms of their design, the population, the duration of the treatment, the final outcome, as well as the variables that could modify the effect, in such a way that there is clinical homogeneity (13). On the other hand, validity depends on a series of concepts and assumptions such as: *transitivity* and *consistency* or *coherence* (16-18). The first one refers to supposing that if intervention B is better than A and intervention A is better than C, then B is better than C. The second one refers to the level of agreement between direct and indirect comparisons.

The t*ransitivity* and *consistency* conditions should be evaluated in all network meta-analysis, however, t*ransitivit*y should be specially evaluated in indirect comparisons (6).

There are some conditions to determine transitivity (13):

1. The common comparator must be similarly defined when it appears in direct comparisons A versus B and B vs. C. Sometimes a certain flexibility can be allowed, although this must be supported by literature.
2. In those studies, that have no arm or intervention C, it is assumed that the absent arms are due to chance. Transitivity will not be met if the choice of the comparator is associated with the relative efficacy of the interventions.
3. Studies with direct comparisons A vs B and B vs C, do not differ with respect to the distribution of possible modifying variables of the effect. In the assumption that there are new and old treatments, in which some variables may change over time, these could be effect modifiers.
4. Patients randomized in direct comparisons could be assigned to any of the treatments (A, B or C).

*Consistency,* on the other hand, assumes that direct and indirect evidence are estimates of the same parameter. That is, if the additional arm had been included in the experiments A vs B and B vs C, the estimate of the effect should have been similar. There should be no discrepancies among the effect of treatments between direct and indirect comparisons (indirect CA = direct CA) (14-18).

*Consistency* can be evaluated and verified through statistical tests using different statistical tests such as: Bucher method or inconsistency factors (5, 14, 18).

Network meta-analysis not only shows a numerical result; it can also show a qualitative component that allows to show gaps in research for the generation of new ideas. It might also evaluates the presence of biases, for example, reporting and publication biases, as well as suggesting subgroups analysis (19).

This novel statistical technique could estimate a ranking or classification of treatments according to the probability of being the best or most effective intervention. This is determined by a concept called SUCRA (Surface Under the Cumulative Ranking Curve) (20). This type of organization offers the clinician a better way of interpreting the results, to be applied to patients. However, before focusing on this aspect, a solid structure of the network and an appropriate sys-tematic review should be considered to be able to trust the findings (4, 8).

### Writing the Network Meta-analysis

The actual recommendation is based on an actualization of the conventional PRISMA for systematic reviews that involve network meta-analyses, and so it has been named PRISMA-NMA. This checklist-model consists of 32 items, and this tool will allow an adequate report of this new statistical method, given some fundamental points (Table-1) (21-23).

**Table 1:** PRISMA NMA Checklist (23).

Title	
Title	Identify the report as a systematic review incorporating a network meta-analysis (or related form of meta-analysis).
Abstract	
Structured summary	Provide a structured summary including, as applicable: Background: main objectives Methods: data sources; study eligibility criteria, participants, and interventions; study appraisal; and synthesis methods, such as network meta-analysis. Results: number of studies and participants identified; summary estimates with corresponding confidence/credible intervals; treatment rankings may also be discussed. Authors may choose to summarize pairwise comparisons against a chosen treatment included in their analyses for brevity. Discussion/Conclusions: limitations; conclusions and implications of findings. Others: primary source of funding; systematic review registration number with registry name.
Introduction	
Rationale	Describe the rationale for the review in the context of what is already known, including mention of why a network meta-analysis has been conducted.
Objectives	Provide an explicit statement of questions being addressed, with reference to participants, interventions, comparisons, outcomes, and study design (PICOS).
Methods	
Protocol and registration	Indicate whether a review protocol exists and if and where it can be accessed (e.g., Web address); and, if available, provide registration information, including registration number.
Eligibility criteria	Specify study characteristics (e.g., PICOS, length of follow-up) and report characteristics (e.g., years considered, language, publication status) used as criteria for eligibility, giving rationale. Clearly describe eligible treatments included in the treatment network, and note whether any have been clustered or merged into the same node (with justification).
Information sources	Describe all information sources (e.g., databases with dates of coverage, contact with study authors to identify additional studies) in the search and date last searched.
Search	Present full electronic search strategy for at least one database, including any limits used, such that it could be repeated.
Study selection	State the process for selecting studies (i.e., screening, eligibility, included in systematic review, and, if applicable, included in the meta-analysis).
Data collection process	Describe method of data extraction from reports (e.g., piloted forms, independently, in duplicate) and any processes for obtaining and confirming data from investigators.
Data items	List and define all variables for which data were sought (e.g., PICOS, funding sources) and any assumptions and simplifications made.
Geometry of the network	Describe methods used to explore the geometry of the treatment network under study and potential biases related to it. This should include how the evidence base has been graphically summarized for presentation, and what characteristics were compiled and used to describe the evidence base to readers.
Risk of bias within individual studies	Describe methods used for assessing risk of bias of individual studies (including specification of whether this was done at the study or outcome level), and how this information is to be used in any data synthesis.
Summary measures	State the principal summary measures (e.g., risk ratio, difference in means). Also describe the use of additional summary measures assessed, such as treatment rankings and surface under the cumulative ranking curve (SUCRA) values, as well as modified approaches used to present summary findings from meta-analyses.
Planned methods of analysis	Describe the methods of handling data and combining results of studies for each network meta-analysis. This should include, but not be limited to: Handling of multi-arm trials; Selection of variance structure; Selection of prior distributions in Bayesian analyses; and Assessment of model fit.
Assessment of Inconsistency	Describe the statistical methods used to evaluate the agreement of direct and indirect evidence in the treatment network(s) studied. Describe efforts taken to address its presence when found
Risk of bias across studies	Specify any assessment of risk of bias that may affect the cumulative evidence (e.g., publication bias, selective reporting within studies).
Additional analyses	Describe methods of additional analyses if done, indicating which were pre-specified. This may include, but not be limited to, the following: Sensitivity or subgroup analyses; Meta-regression analyses; Alternative formulations of the treatment network; and Use of alternative prior distributions for Bayesian analyses (if applicable).
Results	
Study selection	Give numbers of studies screened, assessed for eligibility, and included in the review, with reasons for exclusions at each stage, ideally with a flow diagram.
Presentation of network structure	Provide a network graph of the included studies to enable visualization of the geometry of the treatment network.
Summary of network geometry	Provide a brief overview of characteristics of the treatment network. This may include commentary on the abundance of trials and randomized patients for the different interventions and pairwise comparisons in the network, gaps of evidence in the treatment network, and potential biases reflected by the network structure.
Study characteristics	For each study, present characteristics for which data were extracted (e.g., study size, PICOS, follow-up period) and provide the citations.
Risk of bias within studies	Present data on risk of bias of each study and, if available, any outcome level assessment.
Results of individual studies	For all outcomes considered (benefits or harms), present, for each study: 1) simple summary data for each intervention group, and 2) effect estimates and confidence intervals. Modified approaches may be needed to deal with information from larger networks.
Synthesis of results	Present results of each meta-analysis done, including confidence/credible intervals. In larger networks, authors may focus on comparisons versus a particular comparator (e.g. placebo or standard care), with full findings presented in an appendix. League tables and forest plots may be considered to summarize pairwise comparisons. If additional summary measures were explored (such as treatment rankings), these should also be presented.
Exploration for inconsistency	Describe results from investigations of inconsistency. This may include such information as measures of model fit to compare consistency and inconsistency models, P values from statistical tests, or summary of inconsistency estimates from different parts of the treatment network.
Risk of bias across studies	Present results of any assessment of risk of bias across studies for the evidence base being studied.
Results of additional analyses	Give results of additional analyses, if done (e.g., sensitivity or subgroup analyses, meta-regression analyses, alternative network geometries studied, alternative choice of prior distributions for Bayesian analyses, and so forth).
Discussion	
Summary of evidence	Summarize the main findings, including the strength of evidence for each main outcome; consider their relevance to key groups (e.g., healthcare providers, users, and policy-makers).
Limitations	Discuss limitations at study and outcome level (e.g., risk of bias), and at review level (e.g., incomplete retrieval of identified research, reporting bias). Comment on the validity of the assumptions, such as transitivity and consistency. Comment on any concerns regarding network geometry (e.g., avoidance of certain comparisons).
Conclusions	Provide a general interpretation of the results in the context of other evidence, and implications for future research.
Funding	
Funding	Describe sources of funding for the systematic review and other support (e.g., supply of data); role of funders for the systematic review. This should also include information regarding whether funding has been received from manufacturers of treatments in the network and/or whether some of the authors are content experts with professional conflicts of interest that could affect use of treatments in the network.

**Abbreviations: PICOS** = population, intervention, comparators, outcomes, study design;

* Text in italics indicates wording specific to reporting of network meta-analyses that has been added to guidance from the PRISMA statement.

† = Authors may wish to plan for use of appendices to present all relevant information in full detail for items in this section.

## TECHNIQUE LIMITATIONS

As systematic reviews and meta-analysis are not a *panacea,* and should be conducted under strict and specific methodology conditions, indirect/mixed-treatment comparisons and network meta-analysis have also some potential risks. For example, both *transitivity* and *consistency* conditions, must be met, however, very frequently, publications using this novel technique, forget to assess and state them. Additionally, it should be said that the methods are still being developed, and so they are still of low statistic value, but they have a very promising future (9, 16, 17, 24).

On the other side, the related methods could under or overestimate the effects of treatments, compared to the evidence that comes from direct comparisons (25-27). Further advances in different statistical techniques are still required, in order to increase the available knowledge and so enhance its generalization and applicability on decision making (6).

## CONCLUSIONS

Systematic reviews and network meta-analysis, constitute a tool that can contribute to clinicians and investigators making decisions regarding patients' treatment.

This new methodology involves conducting an excellent, exhaustive, and rigorous systematic review, that will serve as a source of information and ideas. We suggest further training in these techniques for readers, editors, reviewers, and investigators, in order to improve the quality of publications in biomedical journals.
